# Cryomicroscopy *in situ*: what is the smallest molecule that can be directly identified without labels in a cell?

**DOI:** 10.1039/d2fd00076h

**Published:** 2022-08-01

**Authors:** Christopher J. Russo, Joshua L. Dickerson, Katerina Naydenova

**Affiliations:** a MRC Laboratory of Molecular Biology Francis Crick Avenue Cambridge CB2 0QH UK crusso@mrc-lmb.cam.ac.uk

## Abstract

Electron cryomicroscopy (cryoEM) has made great strides in the last decade, such that the atomic structure of most biological macromolecules can, at least in principle, be determined. Major technological advances – in electron imaging hardware, data analysis software, and cryogenic specimen preparation technology – continue at pace and contribute to the exponential growth in the number of atomic structures determined by cryoEM. It is now conceivable that within the next decade we will have structures for hundreds of thousands of unique protein and nucleic acid molecular complexes. But the answers to many important questions in biology would become obvious if we could identify these structures precisely inside cells with quantifiable error. In the context of an abundance of known structures, it is appropriate to consider the current state of electron cryomicroscopy for frozen specimens prepared directly from cells, and try to answer to the question of the title, both now and in the foreseeable future.

## Abstract 2

Current and future technology for *in situ* electron cryomicroscopy is considered from theoretical and practical viewpoints.

## Graphical abstract

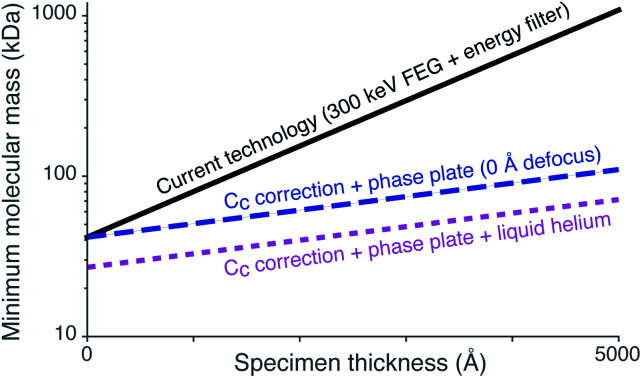

## 1 Introduction

### 1.1 Identifying molecules in micrographs

By far the most successful method of identifying specific molecules within cells is the use of stains and, more recently, molecular tags and labels which generally have greater specificity. These developed naturally from the need to identify specific structures within cells that still could not be seen using the best microscopes of every era, right back to the 19th century. The repertoire of labels includes simple organic dyes, that preferentially bind to specific classes of molecules making them visible by light microscopy and led to the medically important and widely practiced field of histology. A subfield of histology developed alongside the electron microscope that employed stains comprising heavy metals to provide contrast at high resolution in specimens fixed with chemical cross-linking to preserve portions of their native structure for imaging. To this day, many diagnostic tests are still based on the identification of molecules in electron micrographs of chemically fixed specimens taken from tissue biopsies in pathology laboratories around the world. The idea of substituting the “difficult-to-see” biological molecules in a cell with a robust metal that was not, provided the basis for a handful of additional imaging methods, for example cryofixation with freeze substitution,^1^ and freeze fracture/etching^2^ to mention just a couple. All of these were successful in allowing the indirect but high resolution imaging of particular molecules and their arrangement in specific cellular structures, and led to numerous discoveries that now populate the general textbooks of biology. In the context of cell biology and neuroscience, methods using heavy metal stains remain in widespread use and the technology for imaging them continues to be improved, largely through the use of automation of all parts of the specimen preparation and imaging process allowing the indirect imaging of large tissue specimens and even whole organs.^3,4^

Molecular labels have also been used extensively and successfully in the context of electron cryomicroscopy (cryoEM). These include metal nanoparticles,^5–11^ proteins (usually containing metals)^12–15^ or DNA assemblies^16^ for direct identification in electron micrographs, and fluorescent proteins and dyes for correlative fluorescence imaging.^17^ The work on improving and using fluorescent molecular labels at cryogenic temperatures for finding the positions of molecules of interest in electron micrographs and tomograms is reviewed elsewhere.^18^ Currently many of these labelling methods can localise the position of the labelled specimen to within ∼100 Å in some cases, but values >1000 Å are more likely in most instances.

The use of any label has fundamental drawbacks that become increasingly acute at near-atomic resolution. The labels themselves always have the potential to interfere with the structure and function of the molecules being labelled. As often as not, the sensitivity and specificity of the label are less than perfect, meaning not every molecule of interest is labelled, not every label can be seen, and molecules not of interest are incorrectly labelled or detected. Furthermore, as the molecule of interest becomes comparable in size to the tag or linker used to attach it, the localisation precision becomes increasingly problematic. This means that directly imaging the molecules themselves, to the extent that it is possible, is always to be preferred to detecting a surrogate label instead. Of course this is not a useful observation if the signal from the molecule in an image is too small to be detected. But if the resolution revolution^19^ has taught us nothing else, it has shown that the contrast within micrographs can indeed be improved with concerted effort over a number of years. So although we rely on and remain enthusiastic about the use of increasingly sophisticated labels for cryoEM, particularly for the localisation and identification of specific and rare molecules in cryomicrographs, we must also not lose sight of what is possible now and in the foreseeable future without labels as this will remain the preferred option. Then this too will help focus the efforts of those developing new labelling methods and techniques to image them, on that which is clearly impossible by just looking for the molecules themselves. With this in mind, the plethora of atomic structures provided by several decades of structural biology cannot be ignored. In many ways, the atoms themselves can be thought of as the ultimate molecular tags and offer the most hope for mapping out the location and structure of any part of a cell of interest.

### 1.2 Entering an era of plenty in structural biology

**Fig. 1:**
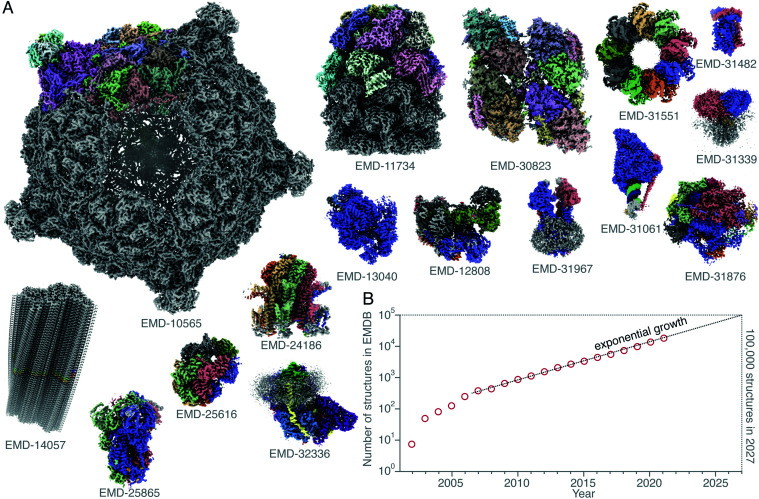
Structures of protein complexes determined by cryoEM. CryoEM maps of all these 16 protein complexes (A) were released in the EMDB^20^ within a single week (10–16 February 2022).^21–36^ The total number of structures released in that week was 121. Here, only some of these structures, whose resolution reached better than 4 Å, and for which atomic models were deposited in the PDB, are shown. All structures are shown on the same scale, and are coloured by chain. The total number of structures in the EMDB is plotted (on a logarithmic scale) *versus* time (B). Currently the number of structures determined by electron microscopy is growing exponentially, and if this trend continues, *ca.* 100 000 new structures of biological molecules and complexes will be published in the next 5 years (2027).

Single-particle electron cryomicroscopy (cryoEM) is an increasingly successful method for determining the structures of purified biological macromolecules (Fig. 1). At least in part, the success of cryoEM is due to the simple yet robust and widely amenable specimen preparation method, introduced by Jacques Dubochet and colleagues some 40 years ago.^37,38^ To this day, most cryoEM specimens for both single-particle electron cryomicroscopy and electron cryotomography (cryo-ET) imaging are prepared using this method. A thin layer of the aqueous specimen is formed on a specimen support (grid), which is then rapidly submerged into a cryogen (usually liquid ethane). Sufficiently fast cooling (<0.1 ms) to <100 K allows for the formation of a vitreous form of solid water, known as low-density amorphous ice. The fast cooling is required to outrun the formation of crystalline ice, and to preserve the biological specimens in a form closely resembling that in solution at ambient temperature, yet in a layer thin enough for electron imaging. Other thick specimens, however, are prepared for imaging by cryoEM using a completely different method: high-pressure freezing (HPF),^1,39,40^ which extends the thickness range into the order of millimetres. Here the specimen is frozen rapidly and under pressure, also to prevent crystallisation. After it is frozen, it is then thinned by some other method. One is using a cryomicrotome to cut slices of the frozen specimen off and apply them to a grid, also to a large extent developed by Dubochet and colleagues,^41,42^ and dubbed CEMOVIS (cryoEM of vitreous sections). A more recent method involves the use of a focused ion beam milling instrument equipped with a cryogenic stage and specimen transfer system,^43–49^ which can be used to thin specimens prepared by either plunge freezing or high-pressure freezing.

The methods for preparing cryoEM specimens, acquiring cryoEM micrographs and tomograms, and processing the data to obtain atomic structures from them have been extensively reviewed,^50–54^ and we will not recapitulate those here. Instead, for the purposes of discussing the future of the field, we wish to consider what are the fundamental physical problems in imaging biological molecules within their native environs, and thus outline our best current understanding of the limits of imaging molecules within cells using electrons. We then look to some technology on the horizon that might push those limits further out and provoke some discussion on the question of the title: what is the smallest molecule that can be directly identified without labels in a cell? In this discussion, we will assume that the atomic structure of the molecule has already been determined by a method such as single-particle cryoEM.

## 2 Phase contrast with cryopreserved cells: where we are and where we want to be

### 2.1 Electron cryotomography: state of the art

**Fig. 2:**
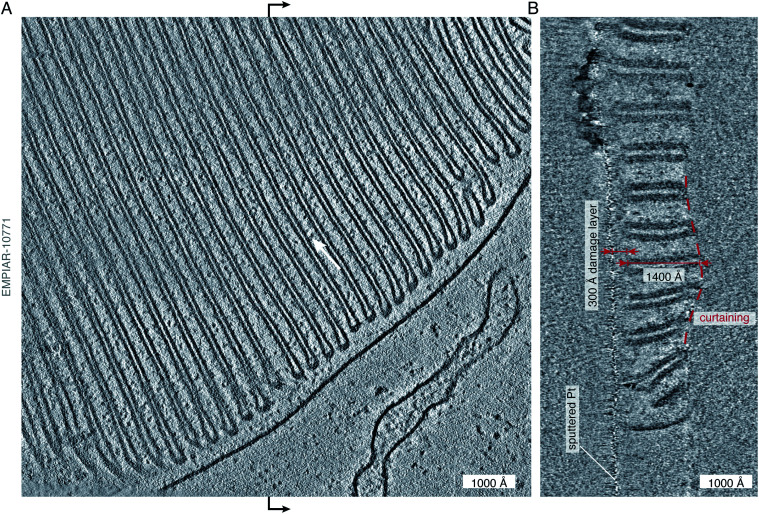
State-of-the art of electron cryotomography of cellular specimens. (A) Central slice through a tomogram of the murine rod outer segment, acquired by the Baumeister group and described in Pöge *et al.*,^56^ demonstrates the impressive quality of state-of-the art tomograms that can be obtained with current technology. The tomogram (EMPIAR-10771) was reconstructed from a tilt series, acquired close to focus (at −2700 Å defocus) using a Volta phase plate.^56^ Individual protein molecules (∼100 kDa in size) are tantalisingly visible between the membranes (white arrow), but their shape, position, and exact identity remain at or below the limit of visibility. (B) Section through the tomogram across the vertical midline (black arrows) of the view in panel (A) shows the profile of the lamella. The curtaining of the lamella, a common artefact of FIB-milling, is highlighted by the dashed red line. The curtain ripple is approximately 400 Å thick and 2000 Å wide. On the other side of the lamella, the platinum sputter coating, applied after milling, as described in Pöge *et al.*,^56^ is visible as the thin line of high-contrast features. The distance between the platinum layer and the first section which shows uncompromised contrast is approximately 300 Å (red arrows), providing an estimate of the thickness of the damage layer on top of the intact lamella created by the FIB. The thickness of the intact lamella is approximately 1400 Å (red arrows), whereas the total thickness of the lamella with the damage layers is 300 + 1400 + 300 = 2000 Å. The damage layer on the other side of the lamella is not as obvious because no platinum coating was applied to that side.

The reductionist approach that structural biology has traditionally taken to understand cellular complexity has proven immensely successful. However, it is becoming increasingly clear that in order to fully understand the complex interactions of molecules that affect biological function, studies of molecular structure must also be performed *in situ*. To fully visualise and understand the complex cellular environment, an integrated approach utilising a wide variety of techniques is necessary. Electron cryotomography is one such technique that combines the high resolving power of a transmission electron microscope with cellular context.^53,55^ In cryoET, a series of tilted micrographs are collected on each imaged area, and these tilted views are reconstructed into a 3D tomogram. This has been particularly useful in visualising large cellular structures^27^ and membrane architecture^56^ (Fig. 2). The tomogram in Fig. 2, taken from the publicly available data published in Pöge *et al.*,^56^ shows the state of the art of *in situ* imaging with electrons. The abundant and organised membrane folds in the rod outer segment are clearly visible in the 3D tomographic reconstruction of a specimen taken from a murine retinal rod cell, which was thinned by focused ion beam (FIB) milling. Low-resolution phase contrast was generated in this example by using a Volta phase plate,^57^ which is an alternative to imaging out of focus. Multiple membrane-associated proteins are visible decorating these folded membranes, but their identity remains difficult to discern. The authors of the study attempt to assign these densities to a putative ∼100 kDa protein complex, but there is just not quite enough information in the tomogram to allow unambiguous identification. The major protein component of the disk membranes, the 38 kDa rhodopsin, which accounts for 90% of the protein content in these membranes, remains indiscernible in the tomogram.

Whilst the tomogram is a beautiful demonstration of the power of cryoET, and its potential for providing direct biological insight, it also gives clues to some of the problems with current methods of *in situ* imaging of thinned cellular specimens. By looking at the lamella in cross section (Fig. 2B), we can see a well demarcated damage layer between the platinum coating on one side of the lamella (which is often applied after milling in order to improve conductivity and reduce charging during imaging) and the region of the lamella where high-resolution structural information is retained. The thickness of this damage layer, created on both sides of the 1400 Å thick lamella by the milling process, is 300 Å (although it is only obvious on the platinum-coated side). This means that all useful structural information comes from a 1400 Å thick volume, contained within 2000 Å of thickness. In addition, we can clearly see the undulations in the surface of the lamella, or curtaining – another common artefact of FIB milling. Finally, outside of the thickness of the reconstructed volume, we see streaking artefacts, which are due to the reconstruction algorithm and lack of information in the direction parallel to the electron beam. In this review we point to some developments that might enable us to go from being able to see the protein ‘dots’ in an electron tomogram, at the edge of what is possible now, to finding their molecular identity with confidence.

To obtain high resolution structural information, molecules of interest in tomograms can be picked and subsequently averaged in a process called subtomogram averaging.^50^ CryoET combined with subtomogram averaging has seen a number of successes, leading to many new insights into the operation of molecules *in situ*. Some examples of the state of the art in this area include the determination of the structure of the HIV capsid proteins,^58^ the structure of the nuclear pore complex in the nuclear membrane^59^ and small molecules bound to ribosomes.^60^ We cite just a handful of recent examples here but refer the reader to other more comprehensive reviews on this topic.^50,61,62^ With improved data processing algorithms, large molecules such as ribosomes can now be visualised using sub-tomogram averaging at resolutions not far behind what can be achieved with single particle cryoEM,^60,63^ albeit with more microscope time and effort required to reach similar resolutions. From the point of view of methods development, in many ways subtomogram averaging can be thought of as a form of single-particle cryoEM analysis using 3D instead of 2D information. An interesting study of vitrified SARS-CoV-2 virions was recently conducted using both single particle methods and sub-tomogram averaging to determine the structure of the spike protein.^64^ In this work it was clear that even with unfettered access to microscopes and specimen preparation tools, the quality and resolution of the structures from single particle cryoEM were superior to those determined by subtomogram averaging from the same specimen. But the tomographic data contributed to understanding the orientation of the spike protein in the membrane. Currently, standard single particle cryoEM without tilt is preferred for structure determination in all cases where it is feasible.

### 2.2 How a thick specimen differs from a thin one

Although cryoET and subtomogram averaging have shown great promise and enabled a wide range of fascinating discoveries, the size of molecules amenable to cryoET still lags well behind that of single particle cryoEM. Thus, *in situ* cryoET studies have mostly been limited to large complexes such as ribosomes. It is a dream of many to be able to directly identify the position and orientation of much smaller proteins and molecules *in situ*. For example, a recent study investigated the distribution of GABA_A_ receptors in synapses, but the capabilities of the state of the art subtomogram averaging were insufficient to discern one pentameric channel type from another or infer its state.^65^ This opens the question: what are the fundamental differences between a single particle specimen and a tomography specimen?

The major difference between them is the thickness of the specimen. Specimens for single particle cryoEM typically have thicknesses of around 300 Å, whereas specimens for cryoET are typically over 1000 Å thick. Given the same molecule of interest, the number of electrons that carry information about the molecule from elastically scattering off it will be the same in a thick or thin sample, but a greater proportion of these are lost to inelastic scattering, and to a lesser extent multiple elastic scattering, in a thick sample. Inelastically scattered electrons have lost a significant amount of energy, meaning that they are overfocused by the objective lens as a result of chromatic aberration. These electrons therefore contribute only noise, which is why energy filtering is often employed to remove them. For a molecule in a 2000 Å thick cell, the signal is less than 40% of that in a 300 Å thick section of a cell.

Other than thickness, it is often harder to get specimens used for cryoET inside the hole of a specimen support. If a supporting layer is necessary, this increases the background signal, and makes it more difficult to prevent beam induced movement of the sample.^66^ As a result, the beam induced motion is often greater for cryoET specimens. Additionally, grids with carbon foils, which are often used for cryoET, suffer from charging and 50 times greater movement than the all-gold grids commonly used for single-particle analysis.^67^ Specimen supports designed specifically for *in situ* imaging may be necessary to overcome some of these problems.

### 2.3 To tilt or not to tilt?

The projection-slice theorem states that the real space 2D projection of a 3D object is equivalent to a central 2D slice of the 3D Fourier transform of that object. To fully populate 3D Fourier space, it is necessary to have projections of the object in multiple orientations, which will correspond to different slices in 3D Fourier space. The Crowther criterion^68^ is a simple equation to determine the minimum number of views, *m*, required to reconstruct a particle of diameter *D*, to a resolution *d*:1
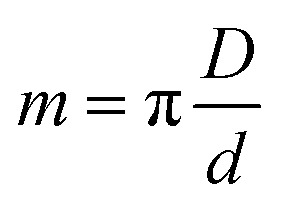


**Fig. 3:**
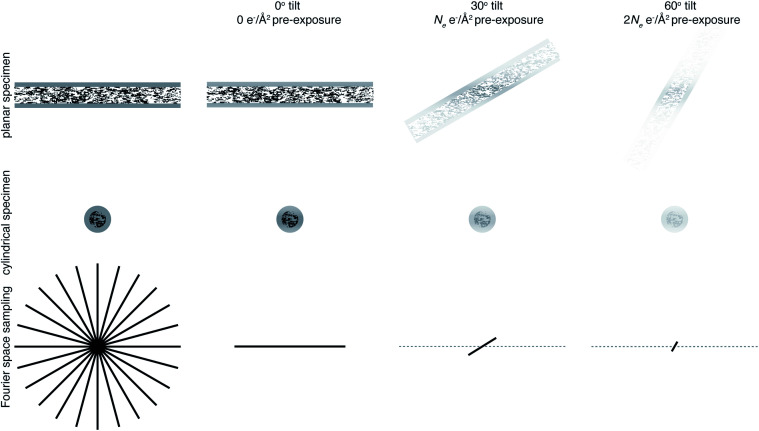
Loss of information at tilt. The top row represents data collection from a planar sample, and the middle row a cylindrical sample geometry. The bottom row is a representation of Fourier space, drawn in 2D for simplicity. Fourier space would ideally be filled isotropically in all directions, shown on the left panel. Using a cylindrical sample geometry aids this by keeping the sample thickness constant as the specimen is tilted. As the sample is irradiated, radiation damage will result in a loss of information, indicated in the top two rows by a fading of the image and in the bottom row by the planes being filled in Fourier space not extending to as high resolution. Later tilt angles will thus be damaged and contain less high resolution information.

For example, reconstructing a molecule of around 300 Å in diameter to 10 Å resolution would require 94 views. If we then consider radiation damage, it quickly becomes clear how challenging obtaining this resolution in all directions is. In the analysis by Henderson,^69^ assuming a critical fluence of 5 e^−^ Å^−2^, it was estimated that 3800 images would be required to obtain 10 Å resolution in all directions. In other words, assuming no radiation damage, it would require a fluence of approximately 200 e^−^ Å^−2^ for each of the 94 projection images. It is immediately clear that this is simply not possible and the sample would be destroyed before even the first image is acquired. Even if the resolution target is much lower, isotropic resolution in all directions is almost impossible as a result of radiation damage. This is illustrated in Fig. 3. As radiation damage progresses, the signal obtained from the same fluence will reduce in every subsequent image. Tilting also introduces further losses of signal not yet discussed. Firstly, for a planar specimen, the effective thickness will increase by a factor of 1/cos *θ*, where *θ* is the tilt angle, leading to an increase in losses from multiple elastic and inelastic scattering. Moreover, as a result of the defocus spread across the field of view that comes with tilting, signal lost to inelastic scattering can no longer be recovered by chromatic aberration correction (Section 3.1). Specimen movement in the plane perpendicular to the electron beam will also be increased^66^ and the specimen charging will likely be worse than in the untilted case. For these reasons, imaging without tilt will always produce higher quality data from radiation sensitive specimens and with higher throughput.^64^ This also means that the decision to have more information in the perpendicular direction must be balanced against a decrease in data quality.

One way in which the negative impact of tilting can be reduced is by having a cylindrical sample geometry.^70^ Since the sample will no longer get thicker as tilt angle increases, higher tilt angles can be used, partially reducing the impact of the missing wedge. The tradeoffs offered by this geometry are just starting to be simulated,^71^ and many are keen to investigate them experimentally. However, as demonstrated in Fig. 3, there will still be a missing wedge where the high resolution signal is reduced by radiation damage in the later tilt angles.

It is clear that Fourier space must be filled in a way other than tilting to get isotropic high resolution in all directions. The way in which this is currently achieved is by combining data from several identical particles in different orientations from multiple tomograms, which is what is done in subtomogram averaging. However, since we are averaging particles in different orientations, what is now the purpose of tilting? In single particle analysis, tilting is often used when there is a problem of preferred orientation.^72^ This could be argued as a reason for tilting in subtomogram averaging. But, if this is the case, the most efficient way to fill Fourier space is to collect data at one (or a few) tilts as well as no tilt, rather than to tilt on the same area. If the purpose of tilting is to prevent molecules of interest from being completely obscured by dense cellular features, then would a tilt pair not suffice for this? If the purpose is to obtain information in the *z* dimension, then it should be possible to perform this more accurately in 2 dimensions using the defocus of the particle. It is clear that tilting will provide low resolution information in *z* not present in a 2D image, so can be useful to give *z* information about large structures such as membranes. The purpose of tilting for subtomogram averaging must therefore be to obtain this rich cellular context along with high resolution structures. Careful thought must be taken to consider exactly how many tilt angles are needed to get cellular context, because each tilt will compromise the data quality. It is possible that many cryoET projects that employ subtomogram averaging could benefit from reducing the number of tilt angles, or possibly even using none whatsoever.^73,74^

### 2.4 Letting go of the projection approximation

The projection approximation, mentioned above and usually assumed at the beginning of any discussion of cryoET, assumes that particles seen in electron micrographs are 2D projections of the 3D molecular density. For images of thin specimens at an accelerating voltage of 300 keV, this approximation largely holds except at very high resolution and for large particles. As specimen thickness increases, the projection approximation begins to break down. Perhaps the most intuitive way for electron microscopists to visualise this is to consider different heights in the specimen as having different defocus values. This intuitive view led to an improvement in the quality of cryoET data processing, by applying different CTF corrections for particles at different heights.^75^ In spite of its success, this method is not necessarily optimal since as the resolution increases, the thickness of the slices must also decrease. It may be time to let go of the projection approximation and treat micrographs of thick specimens differently.

**Fig. 4:**
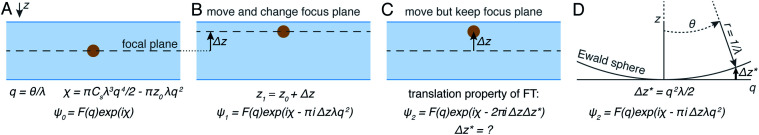
Demonstration of mathematical equivalence between the multiple slices CTF correction and Ewald sphere correction. In (A) the wave scattered off a particle is described as the Fourier transform of the scattering density, multiplied by the contribution of the objective lens. If a particle is moved (B), an extra phase shift can be applied by also shifting the focus plane. If the focus plane is kept the same (C), the translation property of Fourier transforms can be exploited. Using an Ewald sphere construction (D), it can be shown that this is mathematically identical to shifting the defocus plane.

Another way to think about the breakdown of the projection approximation is in terms of the Ewald sphere construction in Fourier space.^76^ In a single image, only the portion of a 3D Fourier transform that lies along the surface of the Ewald sphere is recorded. A 2D projection thus forms a curved plane in 3D Fourier space instead of the flat plane assumed by the projection approximation. Methods have been proposed as to how to correct for the Ewald sphere curvature^77–79^ which have been implemented in several data processing packages.^60,80^ As demonstrated in Fig. 4, the correction of Ewald sphere curvature is mathematically equivalent to moving the plane of focus. It is thus equivalent to using an infinite number of infinitely thin focal slices for CTF correction, but considerably more computationally efficient and accurate.

## 3 How to get there: new technology to improve imaging of thick specimens

### 3.1 Imaging hardware

**Fig. 5:**
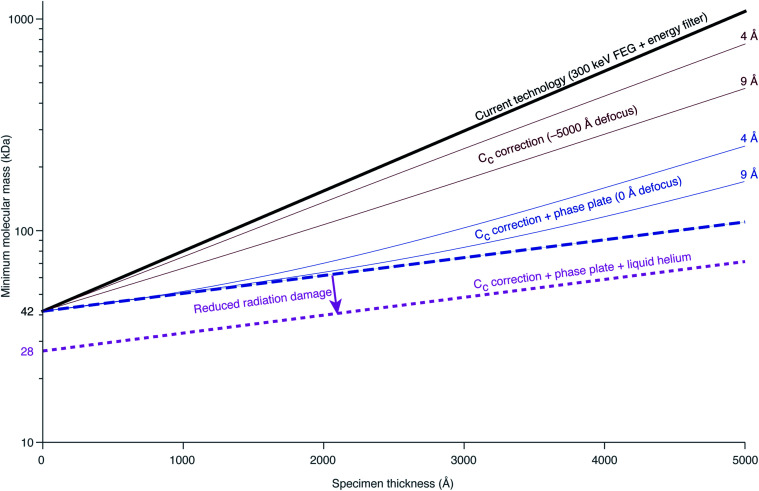
Plots of the minimum protein molecular mass identifiable *in situ* as a function of specimen thickness. The top black line represents the theoretical limit for minimum protein size given current technology. The dashed blue line represents the maximum possible gain from *C*_c_ correction. The amount of gain from *C*_c_ correction is both resolution and defocus dependent. The solid brown lines represent the minimum molecular mass identifiable at a defocus of −5000 Å at 4 and 9 Å resolution. The solid blue lines demonstrate the improvement to this by imaging at 0 defocus (*i.e.* with a phase plate). The lower pink line represents the change in minimum identifiable molecular weight if radiation damage is reduced by a factor of 1.5 from liquid helium cooling.

Richard Henderson famously predicted in 1995, using fundamental scattering cross sections, that the minimum molecular weight of a protein that could be successfully aligned by cryoEM was 38 kDa.^69^ We have recently extended these calculations to thick specimens, and the results are described in Dickerson *et al.*^81^ and plotted in Fig. 5. The upper black line represents the theoretical minimum limit for protein size that we can align *in situ*. This is assuming that the specimen is perfect and there are no other losses in signal. Practically, the targets currently amenable to *in situ* cryoET are limited to large complexes. With the current hardware capabilities, we would not be able to identify sub-100 kDa proteins inside a cell without the use of labels.

**Fig. 6:**
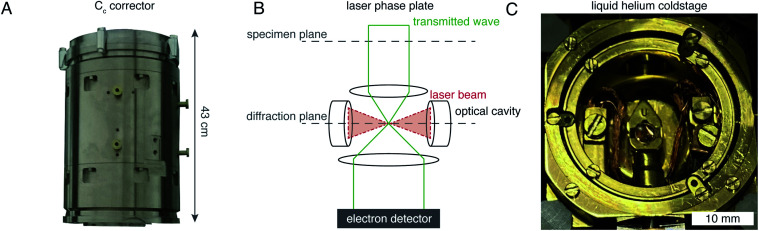
EM hardware developments that could enhance *in situ* imaging. (A) This photograph shows the SALVE *C*_c_/*C*_s_ corrector,^102^ manufactured by CEOS. This particular device is designed for low-voltage (20–80 keV) TEMs, but *C*_c_ correctors for 300 keV microscopes are also manufactured by the same company. (B) The principle of the laser phase plate is diagrammed, after Axelrod *et al.*^103^. (C) This photograph shows a liquid-helium specimen holder from a Tecnai Polara cryomicroscope. The specimen cartridge is surrounded by two concentric layers of shielding, held at liquid-helium (inner) and liquid-nitrogen (outer) temperatures.

There are hardware technologies already in development that could potentially push the theoretical limit further down. One of these is a chromatic aberration (*C*_c_) corrector (Fig. 6A), which compensates for the *C*_c_ in the objective lens, meaning that inelastically scattered electrons can now be correctly focused^82^ instead of being discarded by energy filtering. This means that electrons carrying elastically scattered information in their wavefronts but that have also inelastically scattered can now contribute productively to the signal in the image. However, as a result of specimen-induced decoherence, it is necessary to image in focus to get the maximum benefit of the *C*_c_ corrector.^81^ As a result, even at only −5000 Å of defocus, there is a significant loss of signal, seen by the brown lines in Fig. 5. To image in focus and have enough low-resolution contrast to be able to see cellular context, a mechanism other than defocus is needed for generating phase contrast. Several phase plates have been developed to address this need for low-resolution contrast,^83–85^ but all to date have had problems leading to significant loss of signal.^86^ Designs which omit any solid material susceptible to charging from the beam path, such as the laser phase plate^87,88^ (Fig. 6B) or the obstruction-free anamorphic phase shifter,^89^ could be the solution for phase plates without a loss of signal. Much more work is needed to bring these to practical fruition, particularly in conjunction with a *C*_c_ corrector. By utilising a phase plate in conjunction with a *C*_c_ corrector, there is theoretically a significant gain in signal for specimens between 1000–5000 Å thick, as shown by the blue lines in Fig. 5. Further measurements are required to quantify the exact benefit of using a *C*_c_ corrector on cryogenically preserved biological specimens with thicknesses in this range.

Another piece of technology that might contribute to improving imaging *in situ* is cryogenic specimen stages that reach temperatures lower than that of liquid nitrogen. Recent analysis has demonstrated that for electron crystallography of biological 2D crystals, there is a ∼30% reduction in the rate of radiation damage when the specimen is cooled from 77 K to 13 K.^90^ If the rate of radiation damage could be reduced, then the minimum molecular weight of protein molecule that could be aligned would be reduced for any thickness of specimen, which is denoted by the pink line in Fig. 5. Given that the radiation damage mechanisms are expected to be the same in crystals as they are in hydrated specimens, it is likely that this relationship carries into single particle cryoEM and cryoET, but imaging at low temperatures is notoriously more difficult than diffraction. As a result, cryostages like the one shown in Fig. 6C were developed in the past for cryoEM and have largely been relegated as no practical benefits could be demonstrated.^91–93^ The closest so far to demonstrating an improvement in imaging at lower temperatures was a recent study by Pfeil-Gardiner *et al.*,^94^ where there was no difference in B-factor between 85 K and 17 K, but there was an increase in beam-induced motion at 17 K, which could have caused the lack of improvement. It is also unknown whether or not cooling the specimen further, to temperature closer to the absolute zero, will provide an even greater reduction in radiation damage.

Combining all of these tangible hardware improvements together, it should be possible to identify sub-100 kDa proteins inside a cell, shown in the bottom dotted line in Fig. 5.

It is also worth considering how these relations would scale if we go to higher energies. According to the analysis of Peet *et al.*,^95^ the optimum energy for a 2000 Å thick specimen of amorphous ice is around 600 keV. This is because it maximises the amount of signal from single elastic scattering for a given amount of energy lost to the sample, named the information coefficient. The extent of radiation damage is likely to be proportional to the energy loss given that the nature of the energy loss is also likely to be the same at those two energies; the collision stopping power dominates the cross section for energies below 100 MeV. The improvement in information coefficient on increasing the energy from 300 to 600 keV is on the order of a few percent. There is a second benefit in going to higher energy in terms of reducing the extent of specimen induced decoherence, and therefore reducing the negative impact of defocus on the obtainable signal from inelastically scattered electrons. This improvement is also on the order of a few percent. There are obvious technical challenges in going to high energies, in particular with the development of *C*_c_ correctors and phase plates. As well as technical challenges, there will clearly be a large cost associated with building and operating microscopes for these higher energies, so these benefits must be weighed against the potential costs.

Specimens thicker than 5000 Å become increasingly difficult for phase contrast imaging with electrons, and beyond one micron thickness multiple elastic scattering becomes dominant. For thick specimens in this range, low-dose cryogenic scanning transmission electron cryomicroscopy (cryoSTEM) with a broad probe offers an efficient method for extracting low resolution information.^96^ This method has been extensively developed at the Weizmann Institute^97,98^ and may attract broader usage in the future for imaging cellular specimens without thinning. Variations of cryoSTEM, such as iDPC-STEM^99^ and ptychography,^100^ are also interesting. iDPC-STEM has so far shown the most promise, with a 3.5 Å structure of tobacco mosaic virus having been recently determined using this approach.^101^

### 3.2 Algorithms

Improved reconstruction algorithms for tilted data have been extensively reviewed elsewhere^54^ and are beyond the scope of our discussion here. We only wish to summarise a few points that are important in the context of other technologies in development. First, as discussed above, it is time to give up on the notion of a projected image and include the full model of CTF and Ewald sphere curvature from the very beginning of data processing. These improved models of contrast transfer, which let go of the projection approximation, will need to be incorporated in order to realise high-resolution imaging of thick specimens. Particularly exciting is the use of template matching for identification and localisation of molecules with known structure in 2D micrographs.^104–108^ The incorporation of accurate CTF models, together with improved radiation damage modelling, has the potential to further extend these approaches. Identification of proteins in membranes of whole cells is challenging at present, and is somewhat more feasible on purified liposomes, exosomes, synaptosomes, or isolated membrane fragments. For the more crowded cellular micrographs, 2D template matching could be assisted by membrane segmentation^109^ and membrane subtraction.^110^ Ultimately, we may be able to identify membrane proteins in cells using their atomic structures as templates, and even discern their conformations under a controlled membrane potential.^111–113^ Finally, we note the potential of machine learning for segmentation and annotation of micrographs and tomograms of crowded cellular specimens.^32,114–118^ These are likely to have success analogous to that of the use of machine learning for particle picking in single-particle cryoEM.^119–121^ Much is being done in this area but there will be much more to do, particularly incorporating prior knowledge of many of the common structures, and their conformational variability, that are now known. It is conceivable that we should soon be able to map the locations and orientations of all proteins of known structure, that are larger than the minimal identifiable size, in a micrograph of a thin section of a cell.

## 4 Specimen preparation: the seemingly inscrutable nature of water at low temperature

It is no accident that the seminal review by Jacques Dubochet on cryoEM in 1988 begins with two sections on the nature of water. In his own words, “during their first fifty years of investigation [electron microscopists] put more effort into finding out how to deal with water than into any other electron-microscopical problem”. Our struggle, and appreciation for the beauty of water in biology continues right alongside efforts to improve the methods for imaging the molecules it surrounds.

### 4.1 Movement

Based on our recent work on specimen movement in single-particle cryoEM, we proposed the following two-stage model for the physics of movement of plunge frozen specimens:^66^

(1) During vitrification, a suspended amorphous water layer buckles due to the differential volume change of water relative to the gold support, if its diameter : thickness aspect ratio exceeds the critical value of 11 : 1.

(2) The amorphous ice behaves like an ultraviscous liquid (LDL) when irradiated with electrons.^122^ This renders any buckled films unstable and causes them to move, whereas any flat films remain stable and do not move.

Some specimens for cryoET (small bacterial or archaeal cells, large filaments, phage or virus particles) are prepared in the same way as specimens for single-particle cryoEM – by plunge freezing. Thus, the theory of specimen movement during plunge freezing and under electron irradiation, developed and experimentally demonstrated in Naydenova *et al.*,^66^ also applies to these specimens. From an extension of this theory, it is expected that for a 1000 Å thick specimen the optimal unsupported ice diameter is 1 μm and for 2000 Å it increases to 2 μm. Examples of the buckling of the water ice surrounding plunge frozen cellular specimens can be seen in scanning electron micrographs acquired at oblique viewing angles, such as those shown, for example, in Fig. 8d in Kuba *et al.*^123^ or Fig. 2 in Schaffer *et al.*^124^ Movement-free supports made specifically for cellular specimens can also be produced by an extension of the method described by Naydenova and Russo.^125^ In particular, and to within some practical limits, the foil thickness and hole diameter can be increased as required to match the geometry of the cell under investigation. Ideally, the support foil should be a metal well (probably made of gold) that encompasses the thickness of the cell and has no lateral dimension greater than about 10× the thickness.

### 4.2 Phases of water at cryogenic temperatures

**Fig. 7:**
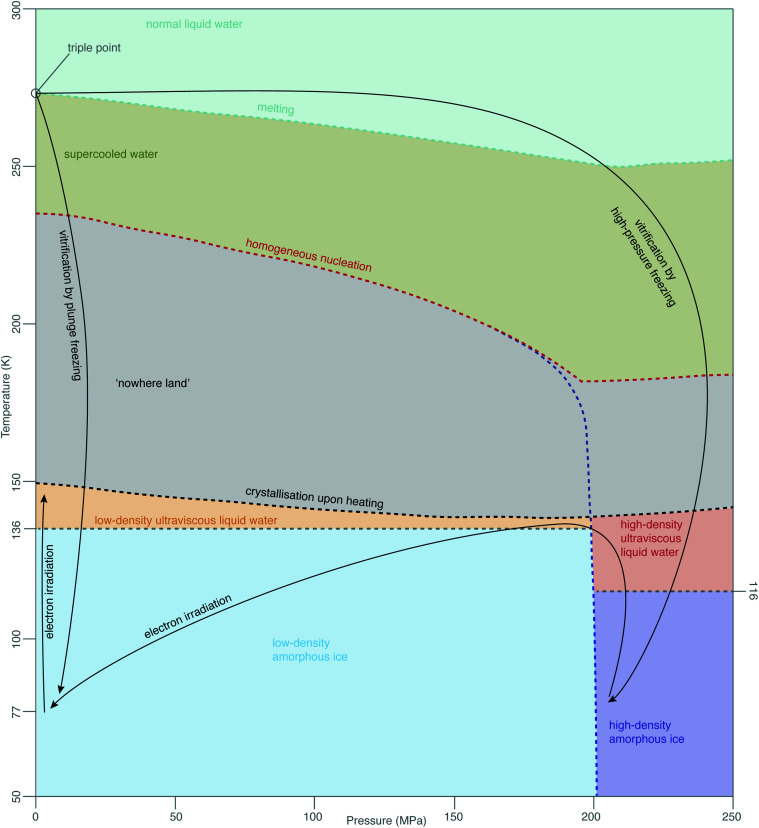
Phases of amorphous water. The diagram of metastable phases of amorphous water at different pressures and temperatures is based on a combination of diagrams from Loerting *et al.*,^126^ Handle *et al.*,^127^ Mallamace *et al.*^128^ The black arrows show the changes, which are most relevant in cryoEM.

While this theory covers specimen motion in plunge-frozen specimens, many fundamental questions about high-pressure frozen amorphous ice remain to be elucidated for *in situ* imaging to reach its full potential. Even the exact phase of the high-pressure frozen ice (low-density amorphous, LDA or high-density amorphous, HDA) is unclear. The typical pressure, applied to the specimen when high-pressure freezing, is around 240 MPa, just enough to enter the HDA zone in the phase diagram in Fig. 7. The specimen is then quenched in liquid nitrogen, ensuring the low temperature preserves the vitreous phase. The properties of HPF ice need to be investigated on the as-prepared specimen, ideally without any further thinning, because the ion beam, typically used for that, might induce further changes to the ice. HDA ice has been studied in protein crystals prepared by HPF, where it was found that upon heating from 80 K to 170 K the phase changed gradually from HDA to LDA, indicating a first-order phase transition.^129^ The transition seems to occur *via* the liquid phases (HDL and LDL), meaning relaxation of stresses in the specimen might be occurring. From the data in Kim *et al.*,^129^ a HPF specimen warmed up to 120 K is expected to convert <20% to LDA. If the phase of amorphous ice prepared by HPF is fully or partially HDA, does electron irradiation change it, and is this phenomenon temperature-dependent? Small flakes of HDA on an amorphous carbon support have been shown to transform into LDA under electron irradiation at around 100 K.^130^ This transformation is believed to occur *via* the corresponding ultraviscous liquid phases (Fig. 7).^131^ How does high-pressure frozen ice (in the configuration of a cryoEM specimen) move under electron irradiation? Are there any stresses built up in the high-pressure frozen ice and what are their causes? Based on the observations from Xu *et al.*,^130^ we expect that these specimens also behave like ultraviscous liquids during irradiation, possibly a mixture of LDL and HDL. LDL and HDL have some different properties, which might further affect the stability of these specimens. For example, somewhat surprisingly, the LDL form is more viscous than HDL.^131,132^ It also remains to be determined how these properties are affected by the composition of the specimen (*e.g.* high concentration of cryoprotectants or proteins).

### 4.3 Amorphous water at liquid-helium temperature

The properties of vitreous ice, prepared by either plunge freezing or HPF, when cooled to 4 K and imaged with electrons at this temperature, also remain controversial. For example, the spacing in the diffuse electron diffraction ring from pure, plunge-frozen vitreous water changes from 3.7 to 3.3 Å after 2–3 e^−^ Å^−2^ irradiation at liquid-helium temperature.^93,133^ One interpretation of these results is that the density of LDA increases by 30% due to a phase transition to a higher density form of amorphous ice, HDA, under irradiation at low temperature. At present, there is no physical mechanism to explain why such a phase transition would occur under these conditions. The only known way to transform LDA to HDA is by compression; HDA is stable relative to LDA only at pressures over 200 MPa (ref. 126) (Fig. 7). Regardless of whether or not there is such a density change, the theory of critical stress for specimen stability^66^ can still be applied. Increased movement of cryoEM specimens has been observed upon cooling to liquid-helium temperature,^91–93,134^ even when all-gold supports are used.^94^ Before we can harness the improvements, afforded by the reduced radiation damage rate at liquid-helium temperature, we will need to find ways to eliminate this problem for the vitreous specimens prepared in all the different ways. While new generations of movement tracking software, especially tailored for tomography, are under active development,^60,120,135^ efforts will continue to physically reduce or eliminate specimen movement, analogous to the recent advances in single-particle cryoEM.^66^

To sum up with another line from Dubochet,^38^ “water, which was once the arch enemy of all electron-microscopists, became what it always was in nature – an integral part of biological matter and a beautiful substance”. The more we delve to understand the behaviour of molecules in water at low temperatures during imaging, the more this rings true, even four decades later.

### 4.4 Radiation damage from high-energy ions

The use of focused ion beams to mill thin lamellae is one of the key techniques available to make specimens from cells thin enough for imaging by phase contrast TEM.^44,48,136^ Measurements comparing the depth and penetration of high energy ions and their secondary electrons are needed to understand how vitrified biological specimens are affected by FIB milling with various ions at different energies. In addition, it is not clear whether and how FIB milling affects the phase and the stress state of the various amorphous ices. Empirically, it has been noted that the milled sections are under tensile stress and various stress-relief geometries (such as notches) have been proposed.^137,138^ Understanding the changes that occur in amorphous ice under irradiation with high-energy ions will be instrumental for eliminating artefacts, instabilities and movement in FIB milled sections. Careful comparisons of the difference between ionic species (*e.g.* He^+^*vs.* Ne^+^*vs.* Ar^+^*vs.* Xe^+^*vs.* O^+^*vs.* N^+^) for etching vitreous biological specimens are certainly required. For example, the amorphous layer produced when milling crystalline silicon is 500 Å thick when using 30 keV Ga^+^, and 300 Å with Xe^+^ at the same energy.^139^ Damage studies from materials science, however, do not map directly to biological specimens and especially frozen hydrated specimens. In inorganic materials, it is the implantation of the ions that causes the most damage, and destroys the structure of the material. In addition, an amorphous surface layer is formed on top of the etched material due to damage and redeposition. The effects of the various FIB milling conditions on simple inorganic specimens have been experimentally studied, simulated, and reviewed extensively.^140,141^ The dependence of the thickness of the damage layers in vitreous biological specimens on total dose, ionic species, direction, and energy remains unknown. Increased beam current will be one of the factors involved, but more work is required to determine the practical utility of different ion beam instruments for milling cryoEM specimens.

Why is this important? The damage layer left behind by the milling process increases the effective thickness of the material being imaged. Using the plot in Fig. 5, if a 500 Å damage layer is present on each side of a 2000 Å thick intact lamella, the size of the smallest molecule that can be identified increases by two-fold. Now let's say for the sake of argument that the damage layer is doubled in thickness due to the type of ion beam used. Then the total thickness would go from 2000 Å + 1000 Å to 2000 Å + 2000 Å and the smallest identifiable molecule would then be 5× larger than if there were no damage at all. Revisiting the FIB-milled lamella from Fig. 2, we can estimate that the damage layer from the most commonly used 30 keV Ga^+^ ions is around 300 Å thick (present on both sides of the specimen). We can compare this with the lateral penetration of 30 keV Ga^+^ ions accelerated at a glancing angle (88°) towards a carbon specimen:^142^ from TRIM simulations^143^ the range is 245 Å. How can we reduce this? The same simulations show, that, for example, a 5 keV beam of the same ions at the same incidence has an approximately 3-fold reduced penetration depth (77 Å).^142^ We expect that the thickness of the damage layer is mostly due to penetration of the ions. The secondary electrons that are generated from the interactions of the ions with the specimen have a low energy (in the eV range),^142^ approximately equal to 4(*m*_electron_/*m*_ion_)*E*_ion_. Thus the penetration depth of these electrons further into the specimen would be small. Reducing the energy of the ion beam used for milling is thus an attractive strategy for reducing the thickness of the damage layer. It comes at the expense of other problems, such as reduced resolution of the optics in the ion column, possibly intensifying the need for correction optics, and increased distortions due to charging during milling (buildup of positive charge on the specimen during exposure to the FIB).

Being able to completely remove the damage layers would equate to reducing the total thickness of the lamella in Fig. 2 from 2000 Å to 1400 Å. This, in turn, would improve the available signal by a factor of 1.3–1.4, according to the plot in Fig. 5 and the more detailed measurements in Dickerson *et al.*^81^ This potential improvement is approximately equal in magnitude to the improvements that we expect are available from hardware innovations such as chromatic aberration correction or liquid-helium specimen cooling. Thus more work is needed to understand the relative merits of and pursue different specimen thinning methods.

## 5 Ideal specimens for *in situ* imaging

**Fig. 8:**
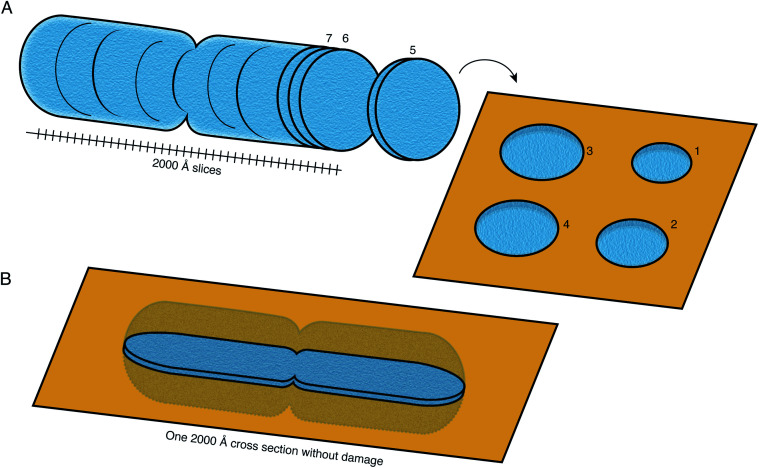
Preparation of sections of cellular specimens with suitable thickness for cryoEM. (A) An ideal specimen preparation method was envisioned since the early days of TEM and dubbed the “most enjoyable dream” of electron microscopists by J. Dubochet.^38^ It would comprise slicing a vitrified whole cell or tissue into a continuum of slices with appropriate thickness for high-resolution TEM imaging (≤2000 Å), and collecting these slices on a support suitable for imaging. In this ideal hypothetical method, no material would be lost or damaged at the interfaces of the slices, such that the entire volume can be imaged from them. No method at present is capable of this. (B) Current sample preparation methods, such as FIB milling, are capable of producing a single slice from the specimen, with thickness suitable for imaging in the TEM. The capability to produce such a slice, but completely undamaged, would already be a major improvement on this method.

FIB milling allows a cellular specimen to be thinned enough to be imaged in the TEM, while ablating the excess material. In the long term, however, this is not the ideal configuration: instead we would like to image all thin sections of the specimen to record its entire three-dimensional volume (Fig. 8). Such a technique is not available at present at the length scales of interest for slicing up cells. While many efforts continue to be focused on developments in FIB milling, we should keep in mind the question: what other methods for thinning down the cryo specimens might be feasible?

### 5.1 Supports tailored for each specimen

Even if we ignore specimen movement, it is clear that current specimen preparation practices for thick specimens can be improved. For example, grids with carbon support foils are still most commonly used in cryoET. It would be advantageous to complement these with a holey gold foil to improve the conductivity of the support and reduce charging, and to improve its mechanical stability. Imaging of unsupported lamellae is compromised by the same charging effects, and can similarly be improved by incorporating a conductive surface into the exposed areas – for example by the use of supporting holey gold foils when preparing specimens for milling. The grid structure itself is also a common cause of problems: for example when cells preferentially adhere to the grid bars, rather than to the foil. Patterning strategies have been developed to direct cell growth away from the gridbars,^144,145^ but this problem could be completely eliminated if the specimen support is re-designed from scratch and the grid bars are omitted entirely. The customisable grid fabrication method, described by Naydenova and Russo,^125^ opens many possibilities for designing supports tailored for the variety of specimens for *in situ* imaging.

### 5.2 The need for good test specimens

Apoferritin has, somewhat accidentally, become the test specimen of choice for single particle cryoEM, much as lysozyme has for X-ray crystallography. In retrospect, the story of how this happened is of some interest since we desire the equivalent for a test specimen for *in situ* imaging. In 2012, after a long conversation at tea in the MRC LMB canteen about many possible proteins to use for testing, one of the authors became convinced that apoferritin would be a good test specimen for technology development and, in particular, for testing grids that may reduce movement of the specimen. The reasons were fourfold: (1) known structure: decades of research from Pauline Harrison and colleagues on ferritin^146,147^ meant there were a wealth of high-resolution structures available in the PDB; (2) identification: even with a quick glance on a phosphor screen, the distinct shape of apoferritin can be identified in a micrograph; (3) wide commercial availability: apoferritin at the time was commercially available from Sigma, (4) high symmetry: thus, in theory, fewer particles are required to calculate the 3D structure of the molecule, but this actually also presents its own challenges in alignment. Apoferritin at the time was indeed a challenge for the single-particle cryoEM data processing software, most of which had been largely developed on other test specimens, such as ribosomes. And so, apoferritin was used to test new specimen supports and was the first biological specimen used to demonstrate the utility of all-gold grids in reducing specimen movement,^148^ because it was at the edge of what was possible with single-particle cryoEM at the time. But it was not until Masa Kikkawa and colleagues in Japan cloned and made freely available the plasmid for bacterial expression of mouse light chain apoferritin,^149^ whose purity and symmetry were superior to the material purified from horse spleens that was commercially available from Sigma, that its use as a test specimen really took off.^149–152^

Given the impending developments in imaging thick specimens, it would now be timely to establish a standard specimen (the ‘apoferritin’) for *in situ* imaging: it could be a very small cell, or it could also be a mixture of proteins with known composition and structure. We note that the standard test specimen can have a substantial and long-term impact on all data processing software that is developed using the data acquired on this specimen. The test specimen that the field selects now for developing *in situ* imaging can in turn influence the capabilities and limitations of the resulting software in future and the outcomes when this software is applied to any other specimens. Multiple bacterial or archaeal cells are thin enough to be imaged in TEM without requiring further thinning, and many examples are shown in the Atlas of Bacterial and Archaeal Cell Structure.^153^ Ideally, the standard specimen should be easy to culture, distribute, reproduce and freeze. If it is a mixture of proteins, they should span a range of masses from what is known to be possible to identify, to what is on the edge and down to what is clearly not possible with current methods – a “molecular ladder”, if you will, for *in situ* imaging.

## 6 Validation and error metrics

Finally, a note of caution: many of us can recall the dangers of blobology and Einstein from noise, which arose from rising expectations in the earlier days of single-particle cryoEM.^154^ Quantifiable error estimates and robust statistical methods will be required to avoid false identification of proteins in micrographs of cellular specimens and incorrect claims regarding resolution and the interpretation of data. New methods for validation, use of randomisation or the omission of data like for the free R-factor^155^ or splitting datasets in half to check for overfitting^156,157^ or similar validation methods will surely be necessary going forward. Real improvements in the imaging of thick specimens are possible, as we have described here, but these will be hard won and require great care in assessing, just as in single-particle cryoEM.

## 7 Conclusions

The potential for imaging myriads of molecules directly within cells is at hand. With a bit of luck and a lot of effort in technological development, it should eventually be possible to identify any individual protein in the 100 kDa mass range in a frozen specimen up to half a micrometer thick, given a known atomic structure of the molecule. For thinner specimens, this limit may be even lower. Hardware development, with long-term international collaboration and robust funding, is essential to realising this potential. Some promising individual elements of electron imaging technology under development that can be put to this task include: chromatic aberration correctors, to bring back the phase contrast from inelastic scattering; new phase plates, which enhance the signal at low spatial frequencies and allow imaging close to focus; and new cryostats at temperatures below the reach of liquid nitrogen to further reduce the effects of radiation damage on the specimen. The improvements in algorithms for processing *in situ* cryoEM data continue, but the need for tilting at all must be re-examined in the context of the wealth of atomic structures available as references; good forward models of the imaging process are also needed to enable even more effective and efficient data processing. It is also likely that the projection approximation will need to be left behind as the data from new electron cryo-microscopes designed for imaging thick specimens improves. Equally important opportunities for improvement are found in the technology for specimen preparation, including the production of thin sections from cells free from damage due to the methods of preparing them. Having robust, inexpensive and accessible specimen supports specifically designed for imaging cells will also be of vital importance as the field matures. We hope that if Michael Faraday were able to attend the Discussions this year, he would be charmed that the careful interplay of electromagnetic fields and forces within an electron microscope is now being used to investigate the subtle nature of so many fascinating molecules.

## Conflicts of interest

There are no conflicts to declare.
